# Evaluating the usefulness of *C5* and *C5AR1* as genetic biomarkers of IgA-mediated vasculitis

**DOI:** 10.1186/s10020-025-01313-3

**Published:** 2025-07-27

**Authors:** Joao Carlos Batista-Liz, Vanesa Calvo-Río, María Sebastián Mora-Gil, María Teresa Leonardo, Ana Cristina Peñalba, Luis Martín-Penagos, Javier Narváez, Belén Sevilla-Pérez, José Luis Callejas-Rubio, Ligia Gabrie, Rafael Gálvez Sánchez, Luis Caminal-Montero, Paz Collado, María José Rodríguez Valls, Diego de Argila, Patricia Quiroga-Colina, Esther Francisca Vicente-Rabaneda, Esteban Rubio, Manuel León Luque, Juan María Blanco-Madrigal, Eva Galíndez-Agirregoikoa, Ricardo Blanco, Verónica Pulito-Cueto, Raquel López-Mejías

**Affiliations:** 1 Immunopathology Group, Fundación Instituto de Investigación Marqués de Valdecilla, Santander, 39011 Spain; 2 Rheumatology Department, Hospital Universitario Marqués de Valdecilla, Santander, 39008 Spain; 3 Division of Paediatrics, Hospital Universitario Marqués de Valdecilla, Santander, 39008 Spain; 4 Division of Nephrology, Hospital Universitario Marqués de Valdecilla, Santander, 39008 Spain; 5 Division of Rheumatology, Hospital Universitario de Bellvitge, L’Hospitalet de Llobregat, 08907 Spain; 6 Division of Paediatrics, Hospital Universitario Clínico San Cecilio, Granada, 18007 Spain; 7 Systemic Autoimmune Disease Unit, Hospital Universitario Clínico San Cecilio, Instituto de Investigación Biosanitaria Ibs.GRANADA, Granada, 18007 Spain; 8 Internal Medicine Department, Hospital Universitario Central de Asturias, Instituto de Investigación Sanitaria del Principado de Asturias (ISPA), Oviedo, 33011 Spain; 9 Division of Rheumatology, Hospital Universitario Severo Ochoa, Leganés, 28914 Spain; 10 Division of Rheumatology, Hospital General de Jerez, Jerez de La Frontera, 11407 Spain; 11 Division of Dermatology, Hospital Universitario de La Princesa, Madrid, 28006 Spain; 12 Division of Rheumatology, IIS-Princesa, Universidad Autónoma de Madrid, Hospital Universitario de La Princesa, Madrid, 28006 Spain; 13 Department of Rheumatology, Hospital Universitario Virgen del Rocío, Sevilla, 41013 Spain; 14 Division of Rheumatology, Hospital Universitario de Basurto, Bilbao, 48013 Spain

**Keywords:** Biomarkers, *C5*, *C5AR1*, Genetics, IgA Vasculitis

## Abstract

**Background:**

IgA-mediated vasculitis (IgAV) is a complex inflammatory disease. Unravelling its genetic background would allow us to identify genetic biomarkers that may be used as additional tools in its daily management, helping to solve the clinical challenge that this vasculitis entails. C5 is a potent immune mediator that is proteolytically processed to generate C5a, a potent anaphylatoxin that exerts its function via C5aR1. *C5* downstream variants (rs3761847 and rs10818488) have been recently related to IgAV pathogenesis. Additionally, C5a and C5aR1 dysregulation contributes to the development of inflammatory diseases, and, particularly, elevated C5a plasma levels have been observed in IgAV patients in the acute stage. Accordingly, we aimed to evaluate the influence of *C5* and *C5AR1* on the pathophysiology of IgAV.

**Methods:**

Eight *C5* (rs10760128, rs74971050, rs4310279, rs7868761, rs10818495, rs10156396, rs3815467, and rs16910280) and three *C5AR1* (rs10853784, rs11673071, and rs11670789) tag variants were genotyped in 342 Caucasian IgAV patients and 723 ethnically matched healthy controls.

**Results:**

No statistically significant differences were observed when *C5* and *C5AR1* frequencies were compared between IgAV patients and healthy controls. Likewise, similar *C5* and *C5AR1* frequencies were observed amongst IgAV patients stratified according to IgAV severity (presence/absence of nephritis). Furthermore, no *C5* and *C5AR1* differences were disclosed when IgAV patients were stratified according to demographic and clinical IgAV characteristics other than nephritis (age at disease onset, presence/absence of joint and gastrointestinal manifestations) and sex.

**Conclusions:**

Our results suggest that *C5* and *C5AR1* are not related to IgAV pathogenesis and, therefore, these genes may not be useful as IgAV genetic biomarkers.

**Supplementary Information:**

The online version contains supplementary material available at 10.1186/s10020-025-01313-3.

## Background

Immunoglobulin A-mediated vasculitis (IgAV) is an immune complex-mediated disease that affects small-sized blood vessels (Jennette et al. 2013). Elevated serum levels of an aberrantly glycosylated galactose-deficient IgA1 (gd-IgA1) underlie IgAV pathogenesis (Suzuki et al. 2018). These increased gd-IgA1 levels lead to glycan-specific IgG antibody development (Suzuki et al. 2009), which forms circulating *gd-IgA1 - IgG anti-gd-IgA1* immune complexes that ultimately deposit on small vessels, activating the complement system, and enhancing neutrophil migration and activation, which results in systemic vascular inflammation and tissue damage (Sugino et al. 2021).

IgAV is described as the most common vasculitis in children (Audemard-Verger et al. 2017; Maritati et al. 2018). This condition may also affect adults, being often associated with worse clinical course and poor outcomes (Blanco et al. 1997; Calvo-Río et al. 2016; Calvo-Río et al. 2014). The classic clinical triad of IgAV encompasses skin, joint, and gastrointestinal (GI) manifestations (Jennette et al. 2013). Additionally, nephritis can also occur in patients with IgAV (Jennette et al. 2013), constituting the most severe feature of the disease as well as its main prognostic factor and the main cause of long-term morbidity and mortality (Pillebout et al. 2002).

This vasculitis represents a major clinical challenge (Abu-Zaid et al. 2021). In this regard, IgAV is mainly diagnosed based on clinical criteria, however relying solely on these criteria has significant limitations and may lead to misdiagnosis or underdiagnosis, especially in patients with atypical skin lesions or in cases where the clinical presentation is incomplete (Ozen et al. 2019; Davin and Weening 2003). Therefore, the detection of IgA1 deposits in blood vessel walls by skin biopsy is currently the most reliable method for confirming the diagnosis of IgAV in clinically suspected cases (Davin and Weening 2003). Nevertheless, it is an invasive procedure that carries risks, especially in paediatric patients, and has significant limitations (Davin and Weening 2003). Also, renal biopsy is the gold standard for the definitive diagnosis of nephritis, although it is an invasive procedure and not suitable for dynamic monitoring (Xu et al. 2022). Additionally, IgAV management is also difficult because of the absence of a correlation between its initial presentation and the long-term renal outcome and the lack of disease-specific therapies (Audemard-Verger et al. 2015; Hernández-Rodríguez et al. 2020). Thus, the identification of sensitive and specific biomarkers could significantly enhance clinical practice by enabling risk prediction, early and definitive diagnosis (including of renal involvement), prognosis, and the development of personalized treatment strategies. Such biomarkers would represent minimally invasive, reproducible, and accessible tools that could help address the current diagnostic, prognostic, and therapeutic challenges associated with IgAV. However, although genetics is crucial in IgAV (López-Mejías et al. 2018; Jelusic and Sestan 2021), no genetic biomarkers are currently available for clinical use in IgAV. To date, our research group along with other authors have described Human Leukocyte Antigen (HLA) *loci *as the main genetic factors involved in IgAV (López-Mejías et al. 2018; López-Mejías et al. 2015; López-Mejías et al. 2015; López-Mejías et al. 2017; Xu et al. 2022). In addition, polymorphisms located in genes mainly related to immune regulation, cytokine and chemokine signalling, adhesion molecules, vascular integrity, and renin-angiotensin system have been also associated with the susceptibility to IgAV and/or the clinical phenotype of this pathology (López-Mejías et al. 2018; He et al. 2013). Therefore, unravelling the genetic background underlying this condition would allow us to identify IgAV genetic biomarkers that may be used as additional tools in its daily management, helping to solve the clinical challenge that IgAV entails.

The complement system is a key element of innate immunity and host defence (Ricklin et al. 2010). Among the proteins comprising this system, C5 is a potent immune mediator (Kurreeman et al. 2010). Genetic variants of *C5* have been associated with several autoimmune diseases, such as systemic lupus erythematosus (Kurreeman et al. 2010; Xu et al. 2013), Behçet’s disease (Xu et al. 2015), and rheumatoid arthritis (Panoulas et al. 2009). Particularly, rs3761847 and rs10818488 (polymorphisms located at *C5* downstream) have been recently associated with IgAV susceptibility and renal damage as well as kidney injury, respectively (Yu et al. 2024). Interestingly, C5 is proteolytically processed to generate C5a, a potent anaphylatoxin that induces increased vascular permeability, chemotaxis of inflammatory cells, activation of several immune cells, cytokine and chemokine release, and phagocytosis (Schanzenbacher et al. 2023; Guo and Ward 2005). This molecule exerts its function via two receptors, being C5aR1 the most widely studied for its ability to bind C5a with high affinity (Bosmann et al. 2012; Karsten et al. 2015). Dysregulated C5a and C5aR1 contribute to the development and progression of multiple immune-mediated diseases (Schanzenbacher et al. 2023; Guo and Ward 2005; Manthey et al. 2009). Remarkably, elevated C5a plasma levels have been observed in IgAV patients in the acute stage, and an effect of this protein on the endothelium of cutaneous small vessels has been proposed (Yang et al. 2015).

Taking all these considerations into account, the main aim of our study was to evaluate for the first time, to our knowledge, the potential influence of *C5* and *C5AR1* on the pathogenesis of IgAV and to determine whether these genes may be useful as genetic biomarkers of the disease using, for that purpose, a large and well-characterized cohort of Caucasian patients with IgAV.

## Materials and Methods

### Study population

Our study, conducted between 2012 and 2024, encompassed a multicentric cohort of 342 unrelated Spanish patients of European ancestry diagnosed with IgAV. All patients fulfilled both Michel et al*.* (Michel et al. 1992) and the American College of Rheumatology (Mills et al. 1990) classification criteria for the diagnosis of IgAV and most of our paediatric patients also fulfilled the EULAR/PRINTO/PRES 2008 classification criteria (Ozen S et al. 2008). The centres involved in the recruitment of these patients included Hospital Universitario Marqués de Valdecilla (Santander), Hospital Universitario de Bellvitge (L'Hospitalet de Llobregat), Hospital Universitario Clínico San Cecilio (Granada), Hospital Universitario Central de Asturias (Oviedo), Hospital Universitario Severo Ochoa (Leganés), Hospital General de Jerez (Jerez de la Frontera), Hospital Universitario de La Princesa (Madrid), Hospital Universitario Virgen del Rocío (Sevilla), and Hospital Universitario de Basurto (Bilbao). Information on the main demographic and clinical features of these patients is shown in Table 1.

**Table 1:** Main demographic and clinical features of the 342 IgAV patients included in the study

Children (≤ 20 years old), % (n)	72.2 (247)
Children age ranges (years, median [minimum– maximum])	7.0 [0.3–20.0]
Adults (> 20 years old), % (n)	27.8 (95)
Adult age ranges (years, median [minimum– maximum])	56.0 [21.0–93.4]
Age at disease onset (years, median [IQR])	7.0 [6.8–29.7]
Duration of follow-up (years, median [IQR])	1.0 [0.2–1.8]
Females/males, % (n)	50.0 (171)/50.0 (171)
Palpable purpura and/or maculopapular rash, % (n)	100.0 (342)
Joint manifestations (if “a” and/or “b”), % (n)	60.2 (206)
a) Arthralgia, % (n)	48.5 (166)
b) Arthritis, % (n)	31.9 (109)
Gastrointestinal manifestations (if “a” and/or “b”), % (n)	52.9 (181)
a) Bowel angina, % (n)	50.3 (172)
b) Gastrointestinal bleeding, % (n)	14.6 (50)
Renal manifestations (if any of the following characteristics), % (n)	37.4 (128)
a) Haematuria^a^, % (n)	33.6 (115)
b) Proteinuria^a^, % (n)	32.2 (110)
c) Nephrotic syndrome^a^, % (n)	5.3 (Jelusic and Sestan 2021)
d) Renal sequelae (persistent renal involvement)^b^, % (n)	7.3 (25)

*IgAV* IgA-mediated vasculitis, *IQR* Interquartile range.

^a^At any time over the clinical course of the disease

^b^At last follow-up

Moreover, 723 unrelated individuals without a history of cutaneous vasculitis or any other autoimmune disease were recruited in our study as healthy controls. These individuals were ethnically matched with IgAV patients and were recruited from the Hospital Universitario Marqués de Valdecilla (Santander) and the National DNA Bank Repository (Salamanca).

### Single nucleotide polymorphisms selection and genotyping method

**Fig. 1:**
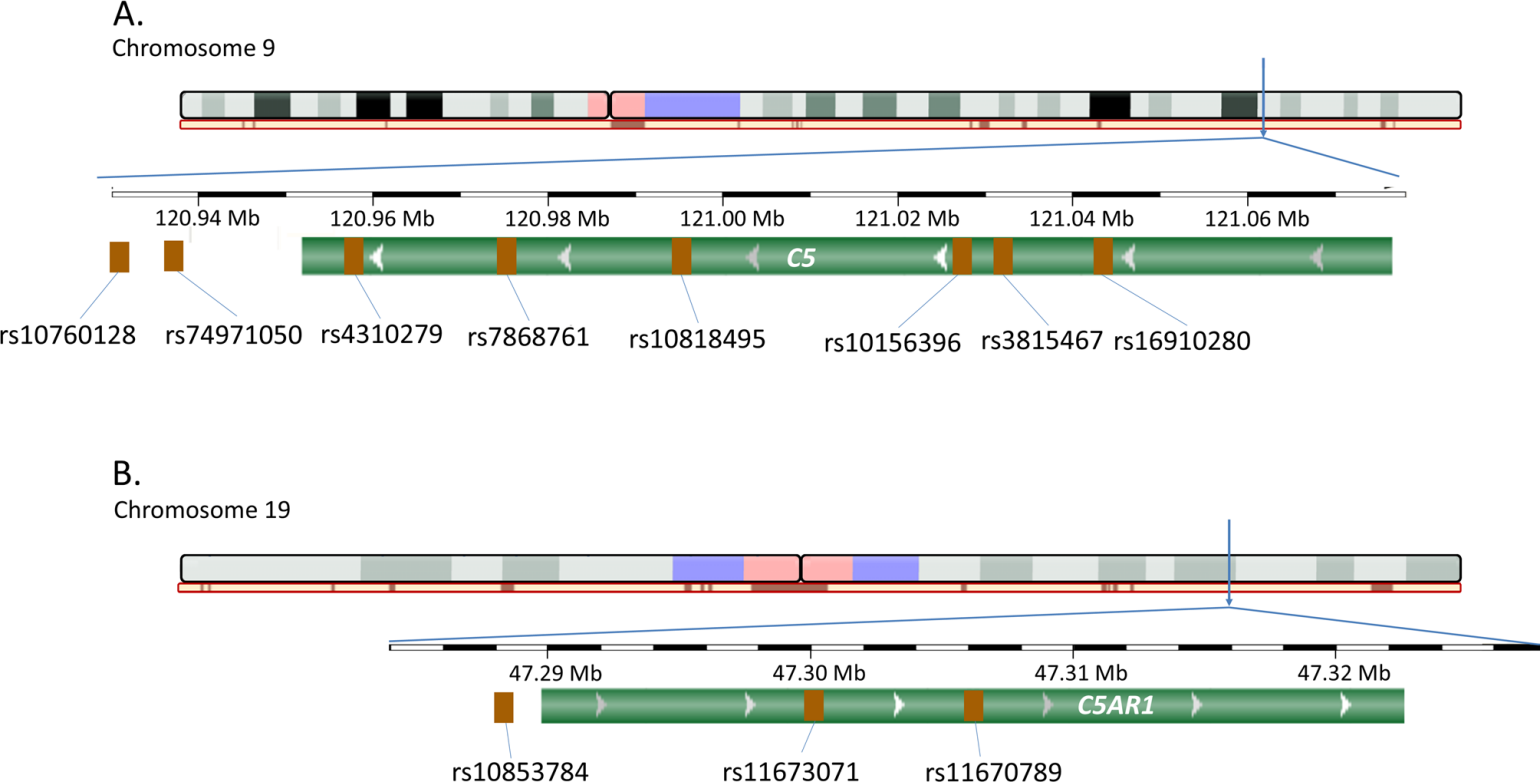
Genomic context of *C5* and *C5AR1* polymorphisms. **A** Genomic context of the *C5* variants evaluated in our study. **B** Genomic context of the *C5AR1* polymorphisms assessed in our study

**Fig. 2:**
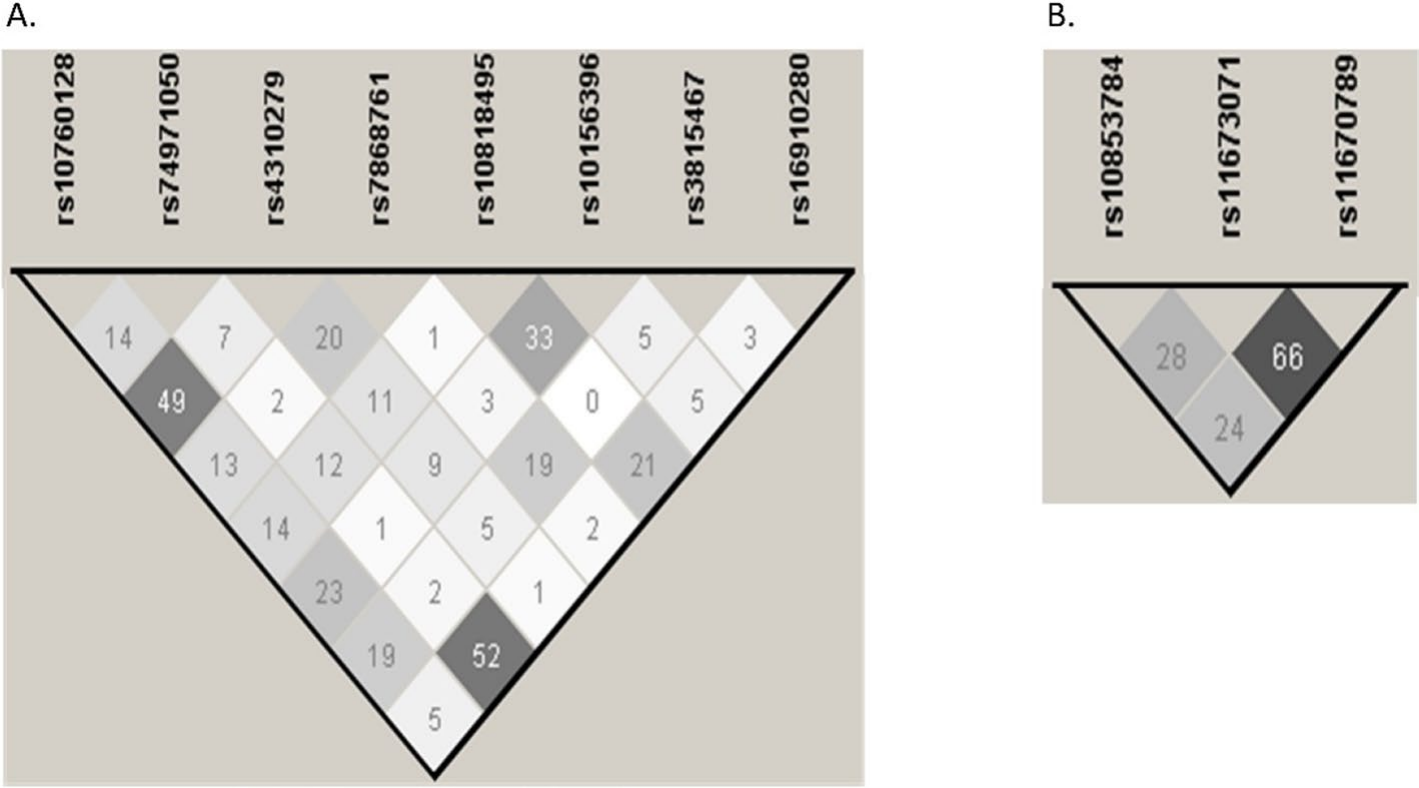
Linkage disequilibrium of *C5* and *C5AR1* polymorphisms in the European population. **A** Linkage disequilibrium of *C5* variants measured by the r^2^ coefficient. Data were obtained by the 1000 Genomes Project and Haploview v.4.2 software, considering the r^2^ threshold set at 0.8 and minimum minor allele frequency at 0.10. The LD amongst the polymorphisms studied is shown on a scale from minimum (white) to maximum (black). **B** Linkage disequilibrium of *C5AR1* polymorphisms measured by the r^2^ coefficient. Data were obtained by the 1000 Genomes Project and Haploview v.4.2 software, considering the r^2^ threshold set at 0.8 and minimum minor allele frequency at 0.10. The LD amongst the polymorphisms studied is shown on a scale from minimum (white) to maximum (black)

Eight tag single nucleotide polymorphisms within *C5* (rs10760128, rs74971050, rs4310279, rs7868761, rs10818495, rs10156396, rs3815467, and rs16910280) and three tag single nucleotide variants within *C5AR1* (rs10853784, rs11673071, and rs11670789), covering most of the variability of both genes, were selected in our study. The genomic context of *C5* and *C5AR1* polymorphisms is displayed in Fig. 1A and B, respectively. Tagging of *C5* and *C5AR1* was performed using data from the 1000 Genomes Project (http://www.internationalgenome.org/) and the Haploview v4.2 software (http://broad.mit.edu/mpg/haploview), and considering the r^2^ threshold set at 0.8 and minimum minor allele frequency at 0.10. The linkage disequilibrium (LD) pattern of *C5* and *C5AR1* variants in the European population is shown in Fig. 2A and B, respectively.

Genomic deoxyribonucleic acid (DNA) from all the individuals included in the study was extracted from peripheral blood using standard procedures. Both patients with IgAV and healthy controls were genotyped for the eleven polymorphisms above mentioned using predesigned TaqMan 5′ single-nucleotide polymorphism genotyping assays (C___2783649_10 for rs10760128, C_101413095_10 for rs74971050, C___2783672_10 for rs4310279, C___2783680_10 for rs7868761, C__30830339_10 for rs10818495, C__30563728_10 for rs10156396, C__25613571_10 for rs3815467, C__32672497_10 for rs16910280, C__11524953_10 for rs10853784, C___1988885_20 for rs11673071, and C__31006669_20 for rs11670789) in a QuantStudioTM 7 Flex Real-Time polymerase chain reaction system, according to the conditions recommended by the manufacturer (Applied Biosystems, Foster City, CA, USA).

To check the accuracy of the genotyping method, both negative controls and duplicate samples were evaluated. Additionally, the genotyping success rate for all the genetic variants included in this study was tested. Furthermore, the deviation of genotype data for the eleven polymorphisms assessed from Hardy–Weinberg equilibrium (HWE) was checked.

### Statistical analyses

Firstly, each *C5* and *C5AR1* polymorphism was analysed independently. Both genotype and allele frequencies of each *C5* and *C5AR1* variant were calculated and compared between patients diagnosed with IgAV and healthy controls. Genotype and allele frequencies of each *C5* and *C5AR1* polymorphism were also compared between patients with IgAV stratified according to the severity of the disease (presence/absence of renal damage), demographic and clinical IgAV characteristics other than nephritis (age at the disease onset and presence/absence of joint and GI manifestations) and sex. The chi-square test or Fisher test (when expected values were below 5) was used for that purpose. The strength of association was estimated using odds ratio (OR) and 95% confidence intervals (CI).

Subsequently, allelic combinations of both *C5* variants and *C5AR1* polymorphisms were carried out. *C5* and *C5AR1* haplotype frequencies were calculated using the Haploview v4.2 software and compared between the groups above mentioned by chi-square test. The strength of association was estimated by OR and 95% CI.

The two-tailed p-values obtained from all the statistical analyses were corrected for multiple testing using the Benjamini–Hochberg method for a False Discovery Rate (FDR) of 5%. P-values less than 0.05 after FDR correction were considered statistically significant.STATA statistical software 12/SE (Stata Corp., College Station, TX, USA) was used to perform all the statistical analyses.

## Results

The genotyping success rate was greater than 98% for all the *C5* (rs10760128, rs74971050, rs4310279, rs7868761, rs10818495, rs10156396, rs3815467, and rs16910280) and *C5AR1* (rs10853784, rs11673071, and rs11670789) polymorphisms.

No deviation from HWE was detected for the eleven variants analysed at the 5% significance level.

The genotype and allele frequencies of each *C5* and *C5AR1* polymorphism in our cohorts of IgAV patients and healthy controls were in accordance with those reported in the 1000 Genomes Project for European populations.

**Fig. 3:**
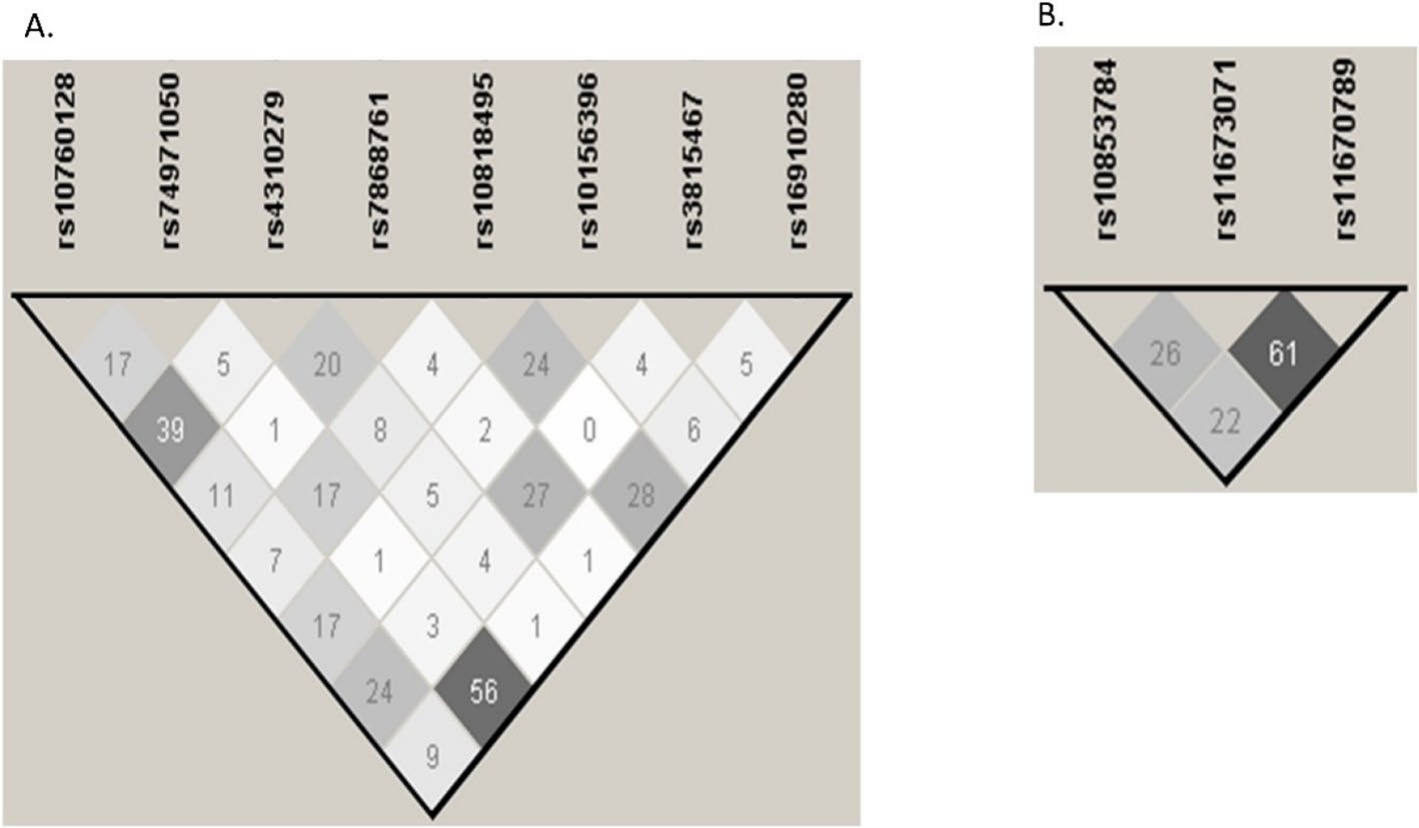
Linkage disequilibrium of *C5* and *C5AR1* polymorphisms in our patients with IgAV I. **A** Linkage disequilibrium of *C5* variants measured by the r^2^ coefficient. Data were obtained by the Haploview v.4.2 software, considering the r^2^ threshold set at 0.8 and minimum minor allele frequency at 0.10. The LD amongst the polymorphisms studied is shown on a scale from minimum (white) to maximum (black). **B** Linkage disequilibrium of *C5AR1* polymorphisms measured by the r^2^ coefficient. Data were obtained by the Haploview v.4.2 software, considering the r^2^ threshold set at 0.8 and minimum minor allele frequency at 0.10. The LD amongst the polymorphisms studied is shown on a scale from minimum (white) to maximum (black)

**Fig. 4:**
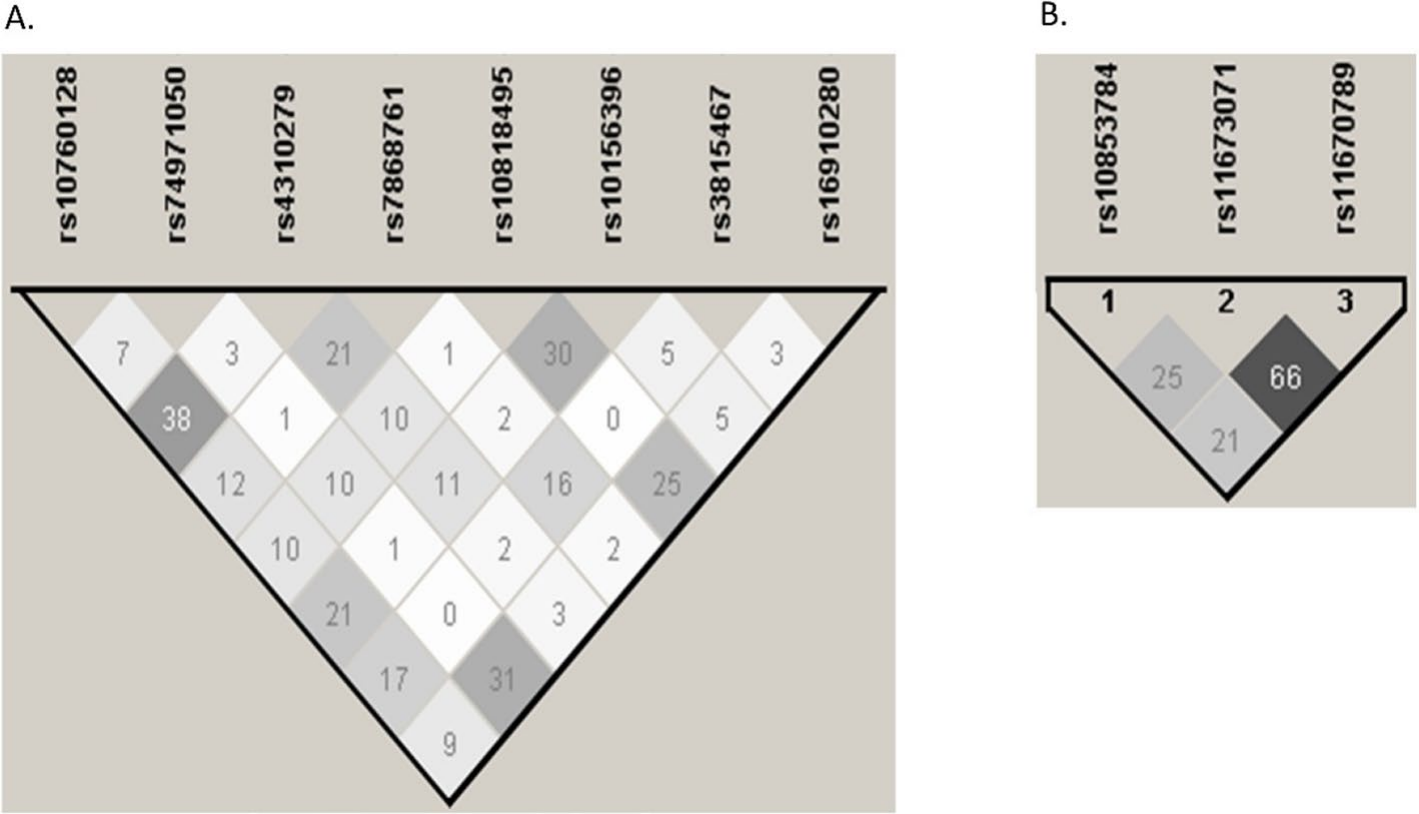
Linkage disequilibrium of *C5* and *C5AR1* polymorphisms in our cohort of healthy controls. **A** Linkage disequilibrium of *C5* variants measured by the r^2^ coefficient. Data were obtained by the Haploview v.4.2 software, considering the r^2^ threshold set at 0.8 and minimum minor allele frequency at 0.10. The LD amongst the polymorphisms studied is shown on a scale from minimum(white) to maximum (black). **B** Linkage disequilibrium of *C5AR1* polymorphisms measured by the r^2^ coefficient. Data were obtained by the Haploview v.4.2 software, considering the r^2^ threshold set at 0.8 and minimum minor allele frequency at 0.10. The LD amongst the polymorphisms studied is shown on a scale from minimum(white) to maximum (black)

The LD of the *C5* and *C5AR1* polymorphisms in our patients with IgAV and our cohort of healthy controls was calculated and in agreement with that observed in the European population (Fig. 3 and Fig. 4, respectively).

### Role of *C5 *and *C5AR1* on IgAV susceptibility

To evaluate the potential influence of *C5* and *C5AR1* on the susceptibility to IgAV, we compared *C5* and *C5AR1* genetic frequencies between patients with IgAV and healthy controls.

Interestingly, when each *C5* polymorphism was assessed independently, no statistically significant differences were observed in the genotype and allele frequencies between IgAV patients and healthy controls (Table 2).

**Table 2:** Genotype and allele analysis of *C5* polymorphisms between IgAV patients and healthy controls

Polymorphism	Genotype, % (n) Allele, % (2n)	IgAV patients	Healthy controls	p	OR [95% CI]	p_FDR_
rs10760128	TT	35.3 (120)	38.7 (278)	-	Ref	-
	TC	49.1 (167)	45.3 (326)	0.24	1.19 [0.89–1.58]	NS
	CC	15.6 (53)	16.0 (115)	0.74	1.07 [0.72–1.58]	NS
	T	59.9 (407)	61.3 (882)	-	Ref	-
	C	40.2 (273)	38.7 (556)	0.51	1.06 [0.88–1.28]	NS
rs74971050	CC	62.4 (212)	63.9 (462)	-	Ref	-
	CT	34.1 (116)	32.4 (234)	0.58	1.08 [0.82–1.42]	NS
	TT	3.5 (12)	3.7 (27)	0.93	0.97 [0.48–1.95]	NS
	C	79.4 (540)	80.1 (1158)	-	Ref	-
	T	20.6 (140)	19.9 (288)	0.72	1.04 [0.83–1.31]	NS
rs4310279	AA	60.8 (208)	65.5 (470)	-	Ref	-
	AG	34.5 (118)	29.9 (214)	0.12	1.25 [0.94–1.65]	NS
	GG	4.7 (16)	4.6 (33)	0.77	1.10 [0.59–2.04]	NS
	A	78.1 (534)	80.5 (1154)	-	Ref	-
	G	21.9 (150)	19.5 (280)	0.20	1.16 [0.93–1.45]	NS
rs7868761	TT	81.2 (277)	83.5 (603)	-	Ref	-
	TC	17.6 (60)	15.5 (112)	0.38	1.17 [0.83–1.65]	NS
	CC	1.2 (4)	1.0 (7)	0.73	1.24 [0.36–4.29]	NS
	T	90.0 (614)	91.3 (1318)	-	Ref	-
	C	10.0 (68)	8.7 (126)	0.35	1.16 [0.85–1.58]	NS
rs10818495	CC	25.7 (88)	30.2 (218)	-	Ref	-
	CA	50.3 (172)	48.6 (351)	0.22	1.21 [0.89–1.65]	NS
	AA	24.0 (82)	21.2 (153)	0.13	1.33 [0.92–1.91]	NS
	C	50.9 (348)	54.5 (787)	-	Ref	-
	A	49.1 (336)	45.5 (657)	0.12	1.16 [0.96–1.39]	NS
rs10156396	CC	65.3 (222)	63.6 (457)	-	Ref	-
	CT	31.2 (106)	31.1 (223)	0.88	0.98 [0.74–1.30]	NS
	TT	3.5 (12)	5.3 (38)	0.20	0.65 [0.33–1.27]	NS
	C	80.9 (550)	79.2 (1137)	-	Ref	-
	T	19.1 (130)	20.8 (299)	0.36	0.90 [0.71–1.13]	NS
rs3815467	GG	70.1 (239)	69.4 (501)	-	Ref	-
	GA	27.3 (93)	28.8 (208)	0.66	0.94 [0.70–1.25]	NS
	AA	2.6 (9)	1.8 (13)	0.40	1.45 [0.61–3.45]	NS
	G	83.7 (571)	83.8 (1210)	-	Ref	-
	A	16.3 (111)	16.2 (234)	0.97	1.01 [0.79–1.29]	NS
rs16910280	CC	59.8 (204)	68.3 (493)	-	Ref	-
	CT	36.4 (124)	27.7 (200)	0.004	1.50 [1.13–1.98]	NS
	TT	3.8 (13)	4.0 (29)	0.82	1.08 [0.55–2.13]	NS
	C	78.0 (532)	82.1 (1186)	-	Ref	-
	T	22.0 (150)	17.9 (258)	0.02	1.30 [1.03–1.62]	NS

*IgAV* IgA-mediated vasculitis, *OR* Odds Ratio, *CI* Confidence interval, *p*_*FDR*_ *p*-values after correcting for multiple testing using the Benjamini–Hochberg method for a False Discovery Rate of 5%, *Ref*. Reference, *NS* Not statistically significant

In addition, similar genotype and allele frequencies of *C5AR1* variants were observed when patients with IgAV were compared to healthy controls (Table 3).

**Table 3:** Genotype and allele analysis of *C5AR1* polymorphisms between IgAV patients and healthy controls

Polymorphism	Genotype, % (n) Allele, % (2n)	IgAV patients	Healthy controls	p	OR [95% CI]	p_FDR_
rs10853784	CC	26.2 (89)	33.3 (239)	-	Ref	-
	CT	54.7 (186)	48.3 (346)	0.02	1.44 [1.07–1.95]	NS
	TT	19.1 (65)	18.4 (132)	0.15	1.32 [0.90–1.94]	NS
	C	53.5 (364)	57.5 (824)	-	Ref	-
	T	46.5 (316)	42.5 (610)	0.09	1.17 [0.98–1.41]	NS
rs11673071	AA	56.4 (193)	53.0 (378)	-	Ref	-
	AG	37.4 (128)	41.1 (293)	0.26	0.86 [0.65–1.12]	NS
	GG	6.2 (21)	5.9 (42)	0.94	0.98 [0.56–1.70]	NS
	A	75.1 (514)	73.6 (1049)	-	Ref	-
	G	24.9 (170)	26.4 (377)	0.44	0.92 [0.75–1.13]	NS
rs11670789	AA	62.3 (212)	58.7 (419)	-	Ref	-
	AG	32.1 (109)	35.6 (254)	0.25	0.85 [0.64–1.12]	NS
	GG	5.6 (19)	5.7 (41)	0.76	0.92 [0.52–1.62]	NS
	A	78.4 (533)	76.5 (1092)	-	Ref	-
	G	21.6 (147)	23.5 (336)	0.33	0.90 [0.72–1.12]	NS

*IgAV* IgA-mediated vasculitis, *OR* Odds Ratio, *CI* Confidence interval, p_FDR_ *p*-values after correcting for multiple testing using the Benjamini–Hochberg method for a False Discovery Rate of 5%, *Ref*. Reference, *NS* Not statistically significant

Furthermore, when all *C5* polymorphisms, as well as *C5AR1* variants, were tested together, conforming haplotypes, no statistically significant differences were observed between IgAV patients and healthy controls (Table 4).

**Table 4:** Haplotype analysis of *C5* and *C5AR1* between IgAV patients and healthy controls

*Locus*	Haplotype, %	IgAV patients	Healthy controls	p	OR [95% CI]	p_FDR_
***C5***	TCATCCGC	35.2	36.1	-	Ref	-
	TTATACGT	16.9	11.9	0.008	1.46 [1.09–1.95]	NS
	CCGTATGC	8.3	8.7	0.89	0.98 [0.67–1.40]	NS
	CCATATGC	7.1	7.3	0.94	0.99 [0.66–1.45]	NS
	CCATCCAC	6.9	6.4	0.58	1.12 [0.74–1.66]	NS
***C5AR1***	TAA	46.0	42.2	-	Ref	-
	CAA	27.0	29.3	0.12	0.84 [0.67–1.05]	NS
	CGG	19.5	21.2	0.16	0.84 [0.65–1.08]	NS

*IgAV* IgA-mediated vasculitis, *OR* Odds Ratio, *CI* Confidence interval, p_FDR_ *p*-values after correcting for multiple testing using the Benjamini–Hochberg method for a False Discovery Rate of 5%, *Ref*. Reference, *NS* Not statistically significant. The table shows the *C5* and *C5AR1* haplotypes with a frequency greater than 5%. Haplotypes are arranged in the following order: *C5* (rs10760128, rs74971050, rs4310279, rs7868761, rs10818495, rs10156396, rs3815467, and rs16910280); *C5AR1* (rs10853784, rs11673071, and rs11670789)

### Influence of *C5* and *C5AR1* on IgAV severity

To determine whether *C5* and *C5AR1* are implicated in the severity of IgAV, we compared their genetic frequencies amongst IgAV patients stratified according to the presence/absence of renal manifestations.

In this respect, genotype and allele frequencies of *C5* variants did not significantly differ between IgAV patients who developed nephritis and those without this complication (Table 5).

**Table 5:** Genotype and allele analysis of *C5* polymorphisms amongst IgAV patients stratified according to the presence/absence of renal manifestations

Polymorphism	Genotype, % (n) Allele, % (2n)	Renal manifestations				
		**Yes**	**No**	**p**	**OR [95% CI]**	**p**_**FDR**_
rs10760128	TT	32.3 (41)	37.1 (79)	-	Ref	-
	TC	50.4 (64)	48.4 (103)	0.47	1.20 [0.73–1.96]	NS
	CC	17.3 (22)	14.6 (31)	0.36	1.37 [0.70–2.67]	NS
	T	57.5 (146)	61.3 (261)	-	Ref	-
	C	42.5 (108)	38.7 (165)	0.33	1.17 [0.85–1.61]	NS
rs74971050	CC	61.4 (78)	62.9 (134)	-	Ref	-
	CT	35.4 (45)	33.3 (71)	0.72	1.09 [0.68–1.74]	NS
	TT	3.2 (4)	3.8 (8)	0.81	0.86 [0.25–2.95]	NS
	C	79.1 (201)	79.6 (339)	-	Ref	-
	T	20.9 (53)	20.4 (87)	0.89	1.03 [0.70–1.51]	NS
rs4310279	AA	61.0 (78)	60.8 (130)	-	Ref	-
	AG	32.0 (41)	36.0 (77)	0.62	0.89 [0.55–1.42]	NS
	GG	7.0 (9)	3.3 (7)	0.14	2.14 [0.76–6.03]	NS
	A	77.0 (197)	78.7 (337)	-	Ref	-
	G	23.0 (59)	21.3 (91)	0.59	1.11 [0.76–1.61]	NS
rs7868761	TT	83.6 (107)	79.8 (170)	-	Ref	-
	TC	16.4 (21)	18.3 (39)	0.60	0.86 [0.48–1.53]	NS
	CC	0.0 (0)	1.9 (4)	0.11	-	NS
	T	91.8 (235)	89.0 (379)	-	Ref	-
	C	8.2 (21)	11.0 (47)	0.23	0.72 [0.42–1.24]	NS
rs10818495	CC	25.8 (33)	25.7 (55)	-	Ref	-
	CA	50.8 (65)	50.0 (107)	0.96	1.01 [0.60–1.72]	NS
	AA	23.4 (30)	24.3 (52)	0.90	0.96 [0.51–1.80]	NS
	C	51.2 (131)	50.7 (217)	-	Ref	-
	A	48.8 (125)	49.3 (211)	0.91	0.98 [0.72–1.34]	NS
rs10156396	CC	63.0 (80)	66.7 (142)	-	Ref	-
	CT	32.3 (41)	30.5 (65)	0.64	1.12 [0.69–1.81]	NS
	TT	4.7 (6)	2.8 (6)	0.33	1.78 [0.55–5.71]	NS
	C	79.1 (201)	81.9 (349)	-	Ref	-
	T	20.9 (53)	18.1 (77)	0.37	1.20 [0.81–1.77]	NS
rs3815467	GG	72.6 (93)	68.5 (146)	-	Ref	-
	GA	25.8 (33)	28.2 (60)	0.56	0.86 [0.52–1.42]	NS
	AA	1.6 (2)	3.3 (7)	0.31	0.45 [0.09–2.22]	NS
	G	85.6 (219)	82.6 (352)	-	Ref	-
	A	14.4 (37)	17.4 (74)	0.32	0.80 [0.52–1.24]	NS
rs16910280	CC	59.4 (76)	60.1 (128)	-	Ref	-
	CT	36.7 (47)	36.1 (77)	0.91	1.03 [0.65–1.63]	NS
	TT	3.9 (5)	3.8 (8)	0.93	1.05 [0.33–3.34]	NS
	C	77.7 (199)	78.2 (333)	-	Ref	-
	T	22.3 (57)	21.8 (93)	0.89	1.03 [0.71–1.49]	NS

*IgAV* IgA-mediated vasculitis, *OR* Odds Ratio, *CI* Confidence interval, p_FDR_ *p*-values after correcting for multiple testing using the Benjamini–Hochberg method for a False Discovery Rate of 5%, *Ref*: Reference, *NS* Not statistically significant

This was also the case when *C5AR1* genotype and allele frequencies were compared amongst IgAV patients with and without renal damage (Table 6).

**Table 6:** Genotype and allele analysis of *C5AR1* polymorphisms amongst IgAV patients stratified according to the presence/absence of renal manifestations

Polymorphism	Genotype, % (n) Allele, % (2n)	Renal manifestations				
		**Yes**	**No**	**p**	**OR [95% CI]**	**p**_**FDR**_
rs10853784	CC	29.1 (37)	24.4 (52)	-	Ref	-
	CT	52.8 (67)	55.9 (119)	0.38	0.79 [0.47–1.33]	NS
	TT	18.1 (23)	19.7 (42)	0.44	0.77 [0.40–1.49]	NS
	C	55.5 (141)	52.4 (223)	-	Ref	-
	T	44.5 (113)	47.7 (203)	0.42	0.88 [0.64–1.20]	NS
rs11673071	AA	57.0 (73)	56.1 (120)	-	Ref	-
	AG	35.2 (45)	38.8 (83)	0.63	0.89 [0.56–1.42]	NS
	GG	7.8 (10)	5.1 (11)	0.38	1.49 [0.60–3.71]	NS
	A	74.6 (191)	75.5 (323)	-	Ref	-
	G	25.4 (65)	24.5 (105)	0.80	1.05 [0.73–1.50]	NS
rs11670789	AA	63.0 (80)	62.0 (132)	-	Ref	-
	AG	30.7 (39)	32.8 (70)	0.73	0.92 [0.57–1.49]	NS
	GG	6.3 (8)	5.2 (11)	0.71	1.20 [0.46–3.12]	NS
	A	78.4 (199)	78.4 (334)	-	Ref	-
	G	21.6 (55)	21.6 (92)	0.99	1.00 [0.69–1.46]	NS

*IgAV* IgA-mediated vasculitis, *OR* Odds Ratio, *CI* Confidence interval, p_FDR_ *p*-values after correcting for multiple testing using the Benjamini–Hochberg method for a False Discovery Rate of 5%, *Ref*. Reference, *NS* Not statistically significant

Besides, the haplotype analysis did not yield additional information, since IgAV patients with nephritis showed similar *C5* and *C5AR1* haplotype frequencies to that obtained in patients who did not suffer renal manifestations (Table 7).

**Table 7:** Haplotype analysis of *C5* and *C5AR1* amongst IgAV patients stratified according to the presence/absence of renal manifestations

*Locus*	Haplotype, %	Renal manifestations				
		**Yes**	**No**	**p**	**OR [95% CI]**	**p**_**FDR**_
***C5***	TCATCCGC	34.5	36.3	-	Ref	-
	TTATACGT	17.4	16.3	0.55	1.15 [0.71–1.86]	NS
	CCGTATGC	9.0	7.9	0.56	1.19 [0.63–2.23]	NS
	CCATATGC	7.2	6.8	0.66	1.15 [0.58–2.27]	NS
	CCATCCAC	7.0	6.7	0.79	1.09 [0.54–2.17]	NS
***C5AR1***	TAA	44.5	46.9	-	Ref	-
	CAA	29.5	25.5	0.30	1.22 [0.82–1.80]	NS
	CGG	20.8	18.6	0.42	1.19 [0.76–1.84]	NS

*IgAV* IgA-mediated vasculitis, *OR* Odds Ratio, *CI* Confidence interval, p_FDR_ *p*-values after correcting for multiple testing using the Benjamini–Hochberg method for a False Discovery Rate of 5%, *Ref*. Reference, *NS* Not statistically significant. The table shows the *C5* and *C5AR1* haplotypes with a frequency greater than 5%. Haplotypes are arranged in the following order: *C5* (rs10760128, rs74971050, rs4310279, rs7868761, rs10818495, rs10156396, rs3815467, and rs16910280); *C5AR1* (rs10853784, rs11673071, and rs11670789)

### *C5* and *C5AR1* impact on demographic and clinical IgAV characteristics other than nephritis and on sex

Finally, to assess the potential relationship between *C5* and *C5AR1* and demographic and clinical characteristics of IgAV other than nephritis and sex, *C5* and *C5AR1* genetic frequencies were compared amongst IgAV patients stratified according to the age at the disease onset, the presence/absence of joint and GI damage, and according to whether IgAV patients were female or male.

No statistically significant differences were observed in the genotype and allele frequencies of each *C5* and *C5AR1* variant between children and adults, nor when IgAV patients with joint or GI complications were compared to those IgAV patients without these manifestations (Additional files 1 and 2, respectively). Also, no *C5* and *C5AR1* genotype or allele differences were found when female IgAV patients were compared to males (Additional files 3 and 4, respectively).

Likewise, *C5* and *C5AR1* haplotype frequencies were similar amongst IgAV patients stratified according to the age at the disease onset, the presence/absence of joint or GI complications, or sex (Additional files 5 and 6, respectively).

## Discussion

This study constituted the first attempt, to our knowledge, to determine whether *C5* and *C5AR1* could be considered useful as IgAV genetic biomarkers. For that purpose, we genotyped eight tag *C5* polymorphisms and three tag *C5AR1* variants in a large and well-characterized cohort of patients with IgAV and European ancestry.

Interestingly, our data evidenced no *C5* and *C5AR1* differences between our IgAV patients and healthy controls, supporting no influence of these genes on IgAV susceptibility. These results are of great interest since there are no previous studies analysing the role of both *C5* and *C5AR1* on the susceptibility to IgAV in Caucasians. To the best of our knowledge, only a previous work performed by Yu et al*.* assessed the involvement of two *C5* downstream *loci* (rs3761847 and rs10818488, both in strong LD with the rs10760128 variant assessed in our study) in IgAV susceptibility, describing a potential effect of *C5* rs3761847 in this regard (Yu et al. 2024). Nevertheless, this study was performed in a cohort of 100 IgAV patients from China (Yu et al. 2024). Consequently, the apparent discrepancies observed between our results and those obtained by Yu et al*.* could be explained by genetic variability in populations of different ethnicities. It is worth mentioning that we examined almost all the variability of *C5* and *C5AR1* in a large and well-characterized cohort of 342 patients diagnosed with IgAV, so our results provide a comprehensive and consistent view of the genetic background of the C5 signalling pathway-mediated inflammation in the susceptibility of IgAV.

Furthermore, no implication of *C5* and *C5AR1* in the development of renal damage was found in our IgAV patients, suggesting that these genes do not represent risk factors for IgAV severity. In this respect, an association between rs3761847 and rs10818488 with kidney injury in Chinese IgAV patients was previously postulated by Yu et al*.* (Yu et al. 2024). Once again, genetic differences between Caucasians and Asians may justify these apparently contradictory results. Interestingly, and in keeping with our data, no relationship between *C5* rs3761847 and inflammatory chronic renal diseases has been previously described in Caucasians (Vuong et al. 2010). Particularly, it is important to underline the lack of association between *C5* rs3761847 and IgA nephropathy (IgAN) (Vuong et al. 2010), an inflammatory chronic renal disease that shares pathogenic and biological abnormalities with IgAV (Xu et al. 2022; Davin et al. 2001; Song et al. 2021). In this context, whether IgAV and IgAN are different pathologies or represent a spectrum of a single disorder is still a matter of debate (Waldos 1988). Accordingly, our findings shed light on this concern since no involvement of *C5* and *C5AR1* with renal damage in IgAV, as previously described in IgAN (Loscalzo et al. 2007), was found in our study, reinforcing the hypothesis that IgAV and IgAN may represent different outcomes of a single disease.

Finally, we disclosed no relationship between *C5* and *C5AR1* and the age at the disease onset, presence/absence of joint and GI damage, and sex in our IgAV patients, indicating that these genes are not related to demographic and clinical IgAV characteristics other than renal manifestations or sex. No previous studies assessing the implication of *C5* and *C5AR1* in these characteristics and sex have been performed so far, highlighting the relevance of our findings in this regard.

The complement system is now considered an attractive therapeutic target for human diseases (Ricklin et al. 2016). Specifically, therapeutic benefits have been proposed for blocking the C5a-C5aR1-axis in several inflammatory disorders (Schanzenbacher et al. 2023), seeming to be especially effective against another systemic small-vessel vasculitis called anti-neutrophil cytoplasmic antibody (ANCA)-associated vasculitis (AAV), where neutrophils become activated by ANCAs. Activated neutrophils then contribute to alternative complement pathway activation, resulting in C5a generation. C5a acts as a potent chemoattractant and activator of neutrophils, amplifying the inflammatory cycle and tissue damage (Kimoto and Horiuchi 2022). Blocking the C5a-C5aR1 axis in AAV has proven to break this cycle by preventing C5a from recruiting and hyper-activating neutrophils which in turn reduces inflammation, limits vessel damage, and halts disease progression (Zipfel et al. 2019; Trivioli and Vaglio 2020). Accordingly, and given that C5a is a potent anaphylatoxin involved in neutrophil recruitment and activation, key processes in the inflammation and tissue damage observed in IgAV, the C5 signalling pathway may play a pivotal role in IgAV pathogenesis. Interestingly, our results show no genetic C5a-C5aR1 implication in IgAV. This is in line with evidence obtained in AAV where the C5a-C5aR1 axis is clearly implicated (Yuan et al. 2012), although genetic variants in *C5* or *C5AR1* have not been associated to the disease to date (Rahmattulla et al. 2016). This suggests that the activation and dysregulation of the C5a–C5aR1 axis in AAV may be driven more by other processes, such as inflammation, rather than by genetic predisposition. Based on these observations, it is plausible to hypothesize that alterations of the C5 signalling pathway implicated in the pathogenesis of IgAV might occur at another biological level beyond genetics and, consequently, functional analysis of the molecules involved in this pathway could provide valuable insights and might identify novel biomarkers for the diagnosis, prognosis and therapeutic monitoring in IgAV.

## Conclusions

In summary, our results suggest that *C5* and *C5AR1* are not related to the susceptibility to IgAV, the severity of the disease, demographic and clinical IgAV characteristics, indicating that these genes may not be useful as genetic biomarkers of IgAV. This study provides new insights into the genetic basis of IgAV, shedding light on its complex aetiology.

## Supplementary Information

Additional file 1. “Genotype and allele analysis of *C5* polymorphisms amongst IgAV patients stratified according to demographic and clinical IgAV characteristics other than renal manifestations”. Table presenting the distribution of *C5* genotypes and alleles among IgAV patients stratified by non-renal demographic and clinical characteristics along with corresponding p-values and odds ratios for statistical associations

Additional file 2. “Genotype and allele analysis of *C5AR1* polymorphisms amongst IgAV patients stratified according to demographic and clinical IgAV characteristics other than renal manifestations”. Table presenting the distribution of *C5AR1* genotypes and alleles among IgAV patients stratified by demographic and non-renal clinical characteristics along with corresponding p-values and odds ratios for statistical associations

Additional file 3. “Genotype and allele analysis of *C5* polymorphisms amongst IgAV patients stratified according to sex”. Table summarizing the distribution of *C5* genotypes and alleles among IgAV patients, stratified by sex along with corresponding p-values and odds ratios for statistical associations

Additional file 4. “Genotype and allele analysis of *C5AR1* polymorphisms amongst IgAV patients stratified according to sex”. Table summarizing the distribution of *C5AR1* genotypes and alleles among IgAV patients, stratified by sex along with corresponding p-values and odds ratios for statistical associations

Additional file 5. “Haplotype analysis of *C5* and *C5AR1* amongst IgAV patients stratified according to demographic and clinical IgAV characteristics other than renal manifestations”. Table presenting the distribution of *C5* and *C5AR1* haplotypes among IgAV patients stratified by demographic and non-renal clinical characteristics along with corresponding p-values and odds ratios for statistical associations

Additional file 6. “Haplotype analysis of *C5* and *C5AR1* amongst IgAV patients stratified according to sex”. Table showing the distribution of *C5* and *C5AR1* haplotypes in IgAV patients, stratified by sex along with corresponding p-values and odds ratios for statistical associations

## Data Availability

The datasets used and/or analysed during the current study are available from the corresponding author on reasonable request.

## Abbreviations

AAV: Anti-neutrophil cytoplasmic antibody associated vasculitis
CI: Confidence interval
DNA: Deoxyribonucleic acid
FDR: False discovery rate
gd-IgA1: Aberrantly glycosylated galactose-deficient IgA1
GI: Gastrointestinal
HWE: Hardy-Weinberg equilibrium
IgAN: Immunoglobulin A nephropathy
IgAV: Immunoglobulin A-mediated vasculitis
LD: Linkage disequilibrium
OR: Odds ratio
