# Supplementary material for: Evaluating the usefulness of C5 and C5AR1 as genetic biomarkers of IgA-mediated vasculitis
Source: Mol Med. 2025 Jul 27;31:267. doi: 10.1186/s10020-025-01313-3 (PMC12296620; doi:10.1186/s10020-025-01313-3)
Supplement: Supplementary file 1 — Additional file 1. “Genotype and allele analysis of C5 polymorphisms amongst IgAV patients stratified according to demographic and clinical IgAV characteristics other than renal manifestations”. Table presenting the distribution of C5 genotypes and alleles among IgAV patients stratified by non-renal demographic and clinical characteristics along with corresponding p-values and odds ratios for statistical associations [file 10020_2025_1313_MOESM1_ESM.docx]

| **Additional file 1.**  Genotype and allele analysis of *C5* polymorphisms amongst IgAV patients stratified according to demographic and clinical IgAV characteristics other than renal manifestations. | | | | | | | | | | | | | | | | |
| --- | --- | --- | --- | --- | --- | --- | --- | --- | --- | --- | --- | --- | --- | --- | --- | --- |
| **Polymorphism** | **Genotype, % (n)**  **Allele, % (2n)** | **Age at the disease onset** | | | | | **Joint manifestations** | | | | | **Gastrointestinal manifestations** | | | | |
|  |  | **Children^a^** | **Adults^b^** | **p** | **OR [95% CI]** | **p_FDR_** | **Yes** | **No** | **p** | **OR [95% CI]** | **p_FDR_** | **Yes** | **No** | **p** | **OR [95% CI]** | **p_FDR_** |
| rs10760128 | TT | 35.5 (87) | 34.7 (33) | - | Ref. | - | 35.8 (73) | 34.6 (47) | - | Ref. | - | 32.2 (58) | 38.7 (62) | - | Ref. | - |
|  | TC | 50.6 (124) | 45.3 (43) | 0.74 | 1.09 [0.64-1.86] | NS | 46.6 (95) | 52.9 (72) | 0.50 | 0.85 [0.53-1.37] | NS | 51.1 (92) | 46.9 (75) | 0.26 | 1.31 [0.82-2.10] | NS |
|  | CC | 13.9 (34) | 20.0 (19) | 0.27 | 0.68 [0.34-1.36] | NS | 17.6 (36) | 12.5 (17) | 0.37 | 1.36 [0.69-2.71] | NS | 16.7 (30) | 14.4 (23) | 0.32 | 1.39 [0.72-2.68] | NS |
|  | T | 60.8 (298) | 57.4 (109) | - | Ref. | - | 59.1 (241) | 61.0 (166) | - | Ref. | - | 57.8 (208) | 62.2 (199) | - | Ref. | - |
|  | C | 39.2 (192) | 42.6 (81) | 0.41 | 0.87 [0.62-1.22] | NS | 40.9 (167) | 39.0 (106) | 0.61 | 1.09 [0.79-1.49] | NS | 42.2 (152) | 37.8 (121) | 0.24 | 1.20 [0.88-1.64] | NS |
| rs74971050 | CC | 60.6 (149) | 67.0 (63) | - | Ref. | - | 62.9 (129) | 61.5 (83) | - | Ref. | - | 61.3 (111) | 63.5 (101) | - | Ref. | - |
|  | CT | 36.6 (90) | 27.7 (26) | 0.16 | 1.46 [0.86-2.48] | NS | 35.6 (73) | 31.8 (43) | 0.71 | 1.09 [0.68-1.74] | NS | 37.0 (67) | 30.8 (49) | 0.35 | 1.24 [0.79-1.97] | NS |
|  | TT | 2.8 (7) | 5.3 (5) | 0.38 | 0.59 [0.16-2.47] | NS | 1.5 (3) | 6.7 (9) | 0.01 | 0.21 [0.06-0.83] | NS | 1.7 (3) | 5.7 (9) | 0.07 | 0.30 [0.08-1.17] | NS |
|  | C | 78.9 (388) | 80.8 (152) | - | Ref. | - | 80.7 (331) | 77.4 (209) | - | Ref. | - | 79.8 (289) | 78.9 (251) | - | Ref. | - |
|  | T | 21.1 (104) | 19.2 (36) | 0.57 | 1.13 [0.74-1.73] | NS | 19.3 (79) | 22.6 (61) | 0.29 | 0.82 [0.56-1.19] | NS | 20.2 (73) | 21.1 (67) | 0.77 | 0.95 [0.65-1.37] | NS |
| rs4310279 | AA | 59.9 (148) | 63.1 (60) | - | Ref. | - | 62.1 (128) | 58.8 (80) | - | Ref. | - | 60.2 (109) | 61.5 (99) | - | Ref. | - |
|  | AG | 36.4 (90) | 29.5 (28) | 0.32 | 1.30 [0.77-2.19] | NS | 31.6 (65) | 39.0 (53) | 0.26 | 0.77 [0.48-1.21] | NS | 34.3 (62) | 34.8 (56) | 0.98 | 1.01 [0.64-1.58] | NS |
|  | GG | 3.7 (9) | 7.4 (7) | 0.21 | 0.52 [0.18-1.47] | NS | 6.3 (13) | 2.2 (3) | 0.12 | 2.71 [0.74-9.90] | NS | 5.5 (10) | 3.7 (6) | 0.44 | 1.51 [0.53-4.33] | NS |
|  | A | 78.1 (386) | 77.9 (148) | - | Ref. | - | 77.9 (321) | 78.3 (213) | - | Ref. | - | 77.4 (280) | 78.9 (254) | - | Ref. | - |
|  | G | 21.9 (108) | 22.1 (42) | 0.95 | 0.99 [0.66-1.48] | NS | 22.1 (91) | 21.7 (59) | 0.90 | 1.02 [0.71-1.48] | NS | 22.7 (82) | 21.1 (68) | 0.63 | 1.09 [0.76-1.57] | NS |
| rs7868761 | TT | 80.5 (198) | 83.2 (79) | - | Ref. | - | 80.5 (165) | 82.3 (112) | - | Ref. | - | 84.0 (152) | 78.1 (125) | - | Ref. | - |
|  | TC | 17.9 (44) | 16.8 (16) | 0.77 | 1.10 [0.58-2.06] | NS | 18.5 (38) | 16.2 (22) | 0.59 | 1.17 [0.66-2.09] | NS | 15.5 (28) | 20.0 (32) | 0.25 | 0.72 [0.41-1.26] | NS |
|  | CC | 1.6 (4) | 0.0 (0) | 0.21 | - | NS | 1.0 (2) | 1.5 (2) | 0.70 | 0.68 [0.09-4.91] | NS | 0.5 (1) | 1.9 (3) | 0.23 | 0.27 [0.03-2.69] | NS |
|  | T | 89.4 (440) | 91.6 (174) | - | Ref. | - | 89.8 (368) | 90.4 (246) | - | Ref. | - | 91.7 (332) | 88.1 (282) | - | Ref. | - |
|  | C | 10.6 (52) | 8.4 (16) | 0.40 | 1.29 [0.71-2.31] | NS | 10.2 (42) | 9.6 (26) | 0.77 | 1.08 [0.64-1.81] | NS | 8.3 (30) | 11.9 (38) | 0.12 | 0.67 [0.40-1.11] | NS |
| rs10818495 | CC | 26.3 (65) | 24.2 (23) | - | Ref. | - | 26.2 (54) | 25.0 (34) | - | Ref. | - | 25.4 (46) | 26.1 (42) | - | Ref. | - |
|  | CA | 47.4 (117) | 57.9 (55) | 0.33 | 0.75 [0.42-1.34] | NS | 51.5 (106) | 48.5 (66) | 0.97 | 1.01 [0.60-1.72] | NS | 50.8 (92) | 49.7 (80) | 0.85 | 1.05 [0.63-1.76] | NS |
|  | AA | 26.3 (65) | 17.9 (17) | 0.41 | 1.35 [0.66-2.78] | NS | 22.3 (46) | 26.5 (36) | 0.49 | 0.80 [0.44-1.49] | NS | 23.8 (43) | 24.2 (39) | 0.98 | 1.01 [0.55-1.84] | NS |
|  | C | 50.0 (247) | 53.2 (101) | - | Ref. | - | 51.9 (214) | 49.3 (134) | - | Ref. | - | 50.8 (184) | 50.9 (164) | - | Ref. | - |
|  | A | 50.0 (247) | 46.8 (89) | 0.46 | 1.13 [0.81-1.59] | NS | 48.1 (198) | 40.7 (138) | 0.49 | 0.90 [0.66-1.22] | NS | 49.2 (178) | 49.1 (158) | 0.98 | 1.00 [0.74-1.36] | NS |
| rs10156396 | CC | 65.3 (160) | 65.3 (62) | - | Ref. | - | 66.8 (137) | 62.9 (85) | - | Ref. | - | 65.2 (118) | 65.4 (104) | - | Ref. | - |
|  | CT | 30.6 (75) | 32.6 (31) | 0.80 | 0.94 [0.56-1.56] | NS | 29.3 (60) | 34.1 (46) | 0.38 | 0.81 [0.51-1.30] | NS | 30.4 (55) | 32.1 (51) | 0.83 | 0.95 [0.60-1.51] | NS |
|  | TT | 4.1 (10) | 2.1 (2) | 0.40 | 1.94 [0.41-9.15] | NS | 3.9 (8) | 3.0 (4) | 0.73 | 1.24 [0.36-4.26] | NS | 4.4 (8) | 2.5 (4) | 0.36 | 1.76 [0.51-6.05] | NS |
|  | C | 80.6 (395) | 81.6 (155) | - | Ref. | - | 81.5 (334) | 80.0 (216) | - | Ref. | - | 80.4 (291) | 81.5 (259) | - | Ref. | - |
|  | T | 19.4 (95) | 18.4 (35) | 0.77 | 1.07 [0.69-1.64] | NS | 18.5 (76) | 20.0 (54) | 0.64 | 0.91 [0.62-1.34] | NS | 19.6 (71) | 18.5 (59) | 0.73 | 1.07 [0.73-1.57] | NS |
| rs3815467 | GG | 71.1 (175) | 67.4 (64) | - | Ref. | - | 69.3 (142) | 71.3 (97) | - | Ref. | - | 72.4 (131) | 67.5 (108) | - | Ref. | - |
|  | GA | 26.8 (66) | 28.4 (27) | 0.68 | 0.89 [0.52-1.52] | NS | 28.3 (58) | 25.8 (35) | 0.62 | 1.13 [0.69-1.85] | NS | 25.4 (46) | 29.4 (47) | 0.38 | 0.81 [0.50-1.31] | NS |
|  | AA | 2.0 (5) | 4.2 (4) | 0.24 | 0.46 [0.12-1.77] | NS | 2.4 (5) | 2.9 (4) | 0.82 | 0.85 [0.22-3.27] | NS | 2.2 (4) | 3.1 (5) | 0.54 | 0.66 [0.17-2.53] | NS |
|  | G | 84.5 (416) | 81.6 (155) | - | Ref. | - | 83.4 (342) | 84.2 (229) | - | Ref. | - | 85.1 (308) | 82.2 (263) | - | Ref. | - |
|  | A | 15.5 (76) | 18.4 (35) | 0.35 | 0.81 [0.52-1.26] | NS | 16.6 (68) | 15.8 (43) | 0.79 | 1.06 [0.70-1.61] | NS | 14.9 (54) | 17.8 (57) | 0.31 | 0.81 [0.54-1.22] | NS |
| rs16910280 | CC | 57.7 (142) | 65.2 (62) | - | Ref. | - | 61.0 (125) | 58.1 (79) | - | Ref. | - | 58.0 (105) | 61.9 (99) | - | Ref. | - |
|  | CT | 39.0 (96) | 29.5 (28) | 0.12 | 1.50 [0.89-2.51] | NS | 36.1 (74) | 36.8 (50) | 0.77 | 0.94 [0.59-1.48] | NS | 38.1 (69) | 34.4 (55) | 0.46 | 1.18 [0.75-1.85] | NS |
|  | TT | 3.3 (8) | 5.3 (5) | 0.54 | 0.70 [0.22-2.23] | NS | 2.9 (6) | 5.1 (7) | 0.28 | 0.54 [0.17-1.68] | NS | 3.9 (7) | 3.7 (6) | 0.87 | 1.10 [0.36-3.40] | NS |
|  | C | 77.2 (830) | 80.0 (152) | - | Ref. | - | 79.0 (324) | 76.5 (208) | - | Ref. | - | 77.1 (279) | 79.1 (253) | - | Ref. | - |
|  | T | 22.8 (112) | 20.0 (38) | 0.44 | 1.18 [0.78-1.78] | NS | 21.0 (86) | 23.5 (64) | 0.43 | 0.86 [0.60-1.25] | NS | 22.9 (83) | 20.9 (67) | 0.53 | 1.12 [0.78-1.62] | NS |
| IgAV: IgA-mediated vasculitis; OR: Odds Ratio; ^a^ ≤20 years old; ^b^ >20 years old; CI: confidence interval; p_FDR_: p-values after correcting for multiple testing using the Benjamini-Hochberg method for a False Discovery Rate of 5%; Ref.: reference; NS: not statistically significant. | | | | | | | | | | | | | | | | |
