# Supplementary material for: Evaluating the usefulness of C5 and C5AR1 as genetic biomarkers of IgA-mediated vasculitis
Source: Mol Med. 2025 Jul 27;31:267. doi: 10.1186/s10020-025-01313-3 (PMC12296620; doi:10.1186/s10020-025-01313-3)
Supplement: Supplementary file 4 — Additional file 4. “Genotype and allele analysis of C5AR1 polymorphisms amongst IgAV patients stratified according to sex”. Table summarizing the distribution of C5AR1 genotypes and alleles among IgAV patients, stratified by sex along with corresponding p-values and odds ratios for statistical associations [file 10020_2025_1313_MOESM4_ESM.docx]

| **Additional file 4.** Genotype and allele analysis of *C5AR1* polymorphisms amongst IgAV patients stratified according to sex. | | | | | | |
| --- | --- | --- | --- | --- | --- | --- |
| **Polymorphism** | **Genotype, % (n)**  **Allele, % (2n)** | **Sex** | | | | |
|  |  | **Female** | **Male** | **p** | **OR [95% CI]** | **p_FDR_** |
| rs10853784 | CC | 29.4 (50) | 22.9 (39) | - | Ref. | - |
|  | CT | 51.8 (88) | 57.7 (98) | 0.17 | 0.70 [0.42-1.17] | NS |
|  | TT | 18.8 (32) | 19.4 (33) | 0.39 | 0.76 [0.40-1.45] | NS |
|  | C | 55.3 (188) | 51.8 (176) | - | Ref. | - |
|  | T | 44.7 (152) | 48.2 (164) | 0.36 | 0.87 [0.64-1.17] | NS |
| rs11673071 | AA | 53.8 (92) | 59.1 (101) | - | Ref. | - |
|  | AG | 40.4 (69) | 34.5 (59) | 0.27 | 1.28 [0.82-2.01] | NS |
|  | GG | 5.8 (10) | 6.4 (11) | 0.99 | 1.00 [0.40-2.46] | NS |
|  | A | 74.0 (253) | 76.3 (261) | - | Ref. | - |
|  | G | 26.0 (89) | 23.7 (81) | 0.48 | 1.13 [0.80-1.60] | NS |
| rs11670789 | AA | 62.4 (106) | 62.3 (106) | - | Ref. | - |
|  | AG | 32.3 (55) | 31.8 (54) | 0.94 | 1.02 [0.64-1.62] | NS |
|  | GG | 5.3 (9) | 5.9 (10) | 0.83 | 0.90 [0.35-2.31] | NS |
|  | A | 78.5 (267) | 78.2 (266) | - | Ref. | - |
|  | G | 21.5 (73) | 21.8 (74) | 0.93 | 0.98 [0.68-1.42] | NS |
| IgAV: IgA-mediated vasculitis; OR: Odds Ratio; CI: confidence interval; p_FDR_: p-values after correcting for multiple testing using the Benjamini-Hochberg method for a False Discovery Rate of 5%; Ref.: reference; NS: not statistically significant. | | | | | | |
