# Supplementary material for: Evaluating the usefulness of C5 and C5AR1 as genetic biomarkers of IgA-mediated vasculitis
Source: Mol Med. 2025 Jul 27;31:267. doi: 10.1186/s10020-025-01313-3 (PMC12296620; doi:10.1186/s10020-025-01313-3)
Supplement: Supplementary file 6 — Additional file 6. “Haplotype analysis of C5 and C5AR1 amongst IgAV patients stratified according to sex”. Table showing the distribution of C5 and C5AR1 haplotypes in IgAV patients, stratified by sex along with corresponding p-values and odds ratios for statistical associations [file 10020_2025_1313_MOESM6_ESM.docx]

| **Additional file 6.** Haplotype analysis of *C5* and *C5AR1* amongst IgAV patients stratified according to sex. | | | | | | |
| --- | --- | --- | --- | --- | --- | --- |
| ***Locus*** | **Haplotype, %** | **Sex** | | | | |
|  |  | **Female** | **Male** | **p** | **OR [95% CI]** | **p_FDR_** |
| ***C5*** | TCATCCGC | 38.0 (129) | 32.6 (111) | - | Ref. | - |
|  | TTATACGT | 16.3 (55) | 17.3 (59) | 0.33 | 0.80 [0.50-1.29] | NS |
|  | CCGTATGC | 8.0 (27) | 8.4 (29) | 0.46 | 0.80 [0.43-1.50] | NS |
|  | CCATATGC | 7.5 (26) | 8.1 (28) | 0.46 | 0.80 [0.42-1.51] | NS |
|  | CCATCCAC | 6.0 (20) | 6.4 (22) | 0.46 | 0.78 [0.38-1.59] | NS |
| ***C5AR1*** | TAA | 44.7 (152) | 47.4 (161) | - | Ref. | - |
|  | CAA | 27.9 (95) | 26.1 (89) | 0.51 | 1.13 [0.77-1.65] | NS |
|  | CGG | 20.2 (69) | 18.7 (64) | 0.52 | 1.14 [0.75-1.75] | NS |
| IgAV: IgA-mediated vasculitis; OR: Odds Ratio; CI: confidence interval; p_FDR_: p-values after correcting for multiple testing using the Benjamini-Hochberg method for a False Discovery Rate of 5%; Ref.: reference; NS: not statistically significant. The table shows the *C5* and *C5AR1* haplotypes with a frequency greater than 5%. Haplotypes are arranged in the following order: *C5* (rs10760128, rs74971050, rs4310279, rs7868761, rs10818495, rs10156396, rs3815467, and rs16910280); *C5AR1* (rs10853784, rs11673071, and rs11670789). | | | | | | |
